# Lifting of Federal Visitation Restrictions and COVID-19 Infections in Nursing Homes

**DOI:** 10.1093/geroni/igaf122.1944

**Published:** 2025-12-31

**Authors:** John Bowblis, Shuang Li, Yong-Fang Kuo, Jennifer Heston-Mullins, James Goodwin, Huiwen Xu

**Affiliations:** Miami University, Oxford, Ohio, United States; UTMB Health, The University of Texas Medical Branch, Galveston, Texas, United States; University of Texas Medical Branch, Galveston, Galveston, Texas, United States; Miami University, Oxford, Ohio, United States; UTMB Health, The University of Texas Medical Branch, Galveston, Texas, United States; Emory University, Atlanta, Georgia, United States

## Abstract

Early in the COVID-19 pandemic, the federal government recommended restricting visitors from entering nursing homes (NH) to reduce the spread of the virus. To date, there is limited evidence to suggest that visitation restrictions reduced the spread of the virus. Using the lifting of federal restrictions on visitation in September 2020, this study compared NH COVID-19 infection rates for states that were only subject to the federal restriction on visitation bans to those that lifted their bans earlier. This statistical framework determines whether lifting visitation restrictions lead to increased infection rates, and thus visitation restrictions were effective at reducing the spread of the virus. Our regression analysis found no statistically significant increase in NH infection rates or the community-adjusted infection rates associated with the lifting of the federal visitation restrictions. NH infections rates were closely tied to community infection rate throughout the study period, suggesting that community spread was the driving factor in infections among NH residents. Visitation restrictions took a physical and emotional toll on residents and family members, and limited the ability of visitors to monitor care. Our results suggest that allowing NHs the freedom to choose whether to allow does not necessarily mean that COVID-19 infections will increase. Thus, future policies should focus on providing general guidance that allows NHs to make the decision on when visitation is or is no appropriate for their facility based on their available resources instead of implementing a universal visitation restriction.

